# The Origin of B-cells: Human Fetal B Cell Development and Implications for the Pathogenesis of Childhood Acute Lymphoblastic Leukemia

**DOI:** 10.3389/fimmu.2021.637975

**Published:** 2021-02-17

**Authors:** Thomas R. Jackson, Rebecca E. Ling, Anindita Roy

**Affiliations:** ^1^Department of Paediatrics and MRC Weatherall Institute of Molecular Medicine, University of Oxford, Oxford, United Kingdom; ^2^National Institute for Health Research (NIHR) Oxford Biomedical Research Centre, Oxford, United Kingdom

**Keywords:** B-lymphopoiesis, human fetal, childhood, infant, leukemia, B-ALL, B-cell

## Abstract

Human B-lymphopoiesis is a dynamic life-long process that starts *in utero* by around six post-conception weeks. A detailed understanding of human fetal B-lymphopoiesis and how it changes in postnatal life is vital for building a complete picture of normal B-lymphoid development through ontogeny, and its relevance in disease. B-cell acute lymphoblastic leukemia (B-ALL) is one of the most common cancers in children, with many of the leukemia-initiating events originating *in utero*. It is likely that the biology of B-ALL, including leukemia initiation, maintenance and progression depends on the developmental stage and type of B-lymphoid cell in which it originates. This is particularly important for early life leukemias, where specific characteristics of fetal B-cells might be key to determining how the disease behaves, including response to treatment. These cellular, molecular and/or epigenetic features are likely to change with age in a cell intrinsic and/or microenvironment directed manner. Most of our understanding of fetal B-lymphopoiesis has been based on murine data, but many recent studies have focussed on characterizing human fetal B-cell development, including functional and molecular assays at a single cell level. In this mini-review we will give a short overview of the recent advances in the understanding of human fetal B-lymphopoiesis, including its relevance to infant/childhood leukemia, and highlight future questions in the field.

## Introduction

Unraveling the details of human hematopoietic development during embryogenesis is crucial for both basic and medical science. Relative contributions of different progenitor compartments and downstream lineage specificity vary during human ontogeny. Detailed immunophenotyping of fetal hematopoietic tissues from 6 to 20 weeks post conception (pcw) has identified that a much higher proportion of fetal bone marrow (FBM) cells are B-lymphoid than fetal liver (FL) and adult bone marrow (ABM) (1). In keeping with this, the changing lymphoid/myeloid specification in aging bone marrow has been described (2–4). Secondly, a switch from multipotent to largely oligo/unipotent stem cells is also known to occur between fetal and adult life (5). Thirdly, differences in the proliferative capacity of human fetal and postnatal hematopoietic stem and progenitor cells (HSPC) have been demonstrated using functional and molecular studies, with a marked and progressive increase in stem cell quiescence evident during physiological aging (6–9). In addition, some fetal gene expression programs are inherently oncogenic (10–12), and high mutation rates are seen both in hematopoietic and non-hematopoietic fetal stem cells when compared to postnatal tissues (13, 14). Therefore, understanding how hematopoiesis changes through human ontogeny is crucial if we are to understand the site- and stage-specific variation in HSPC throughout the human lifetime and the role it plays in hematological disorders/diseases.

Fetal hematopoiesis is of particular interest in understanding childhood blood disorders that originate before birth. Significantly all infant leukemia and much of childhood acute lymphoblastic leukemia (ALL) originate before birth (15, 16).

ALL is the most common childhood malignancy, and 80% of childhood-ALL are of the B-lymphoid lineage. Early onset B-ALL can be divided into infant ALL (iALL) presenting at age <12 months or childhood-ALL presenting at age >12 months. While outcomes for childhood-ALL have improved dramatically over the past few years to reach an overall survival (OS) rate of >90% (17); the OS rate is only ~60% in infants (18). The reasons for such disparate outcomes is not clear, but the clues might lie in the developmental origins of infant and childhood-ALL.

Advances in understanding fetal hematopoiesis and prenatal oncogenic events, have been limited by a number of factors. The scarcity of human fetal biological samples is compounded by the difficulty in working with very small numbers of HSPC that can be obtained from each sample. Thus, majority of our understanding of early hematopoiesis development has come from murine studies. Neither these, nor adult human models can be used as a faithful surrogate for human fetal hematopoiesis (5, 19, 20). This in turn leads to difficulties in making developmentally relevant model systems for human leukemia (21, 22).

In this review we will focus on recent advances in our understanding of human B-lymphopoiesis during ontogeny, especially in fetal life, and review progenitor compartments therein which may align to the origin of iALL and childhood-ALL.

## Human B-Lymphopoiesis

Hematopoiesis has traditionally been described as a hierarchical process with hematopoietic stem cells (HSCs) at the apex; these divide and differentiate into progressively restricted progenitors that subsequently give rise to the mature cell types of the hematopoietic and immune system (23, 24).

**Figure 1:**
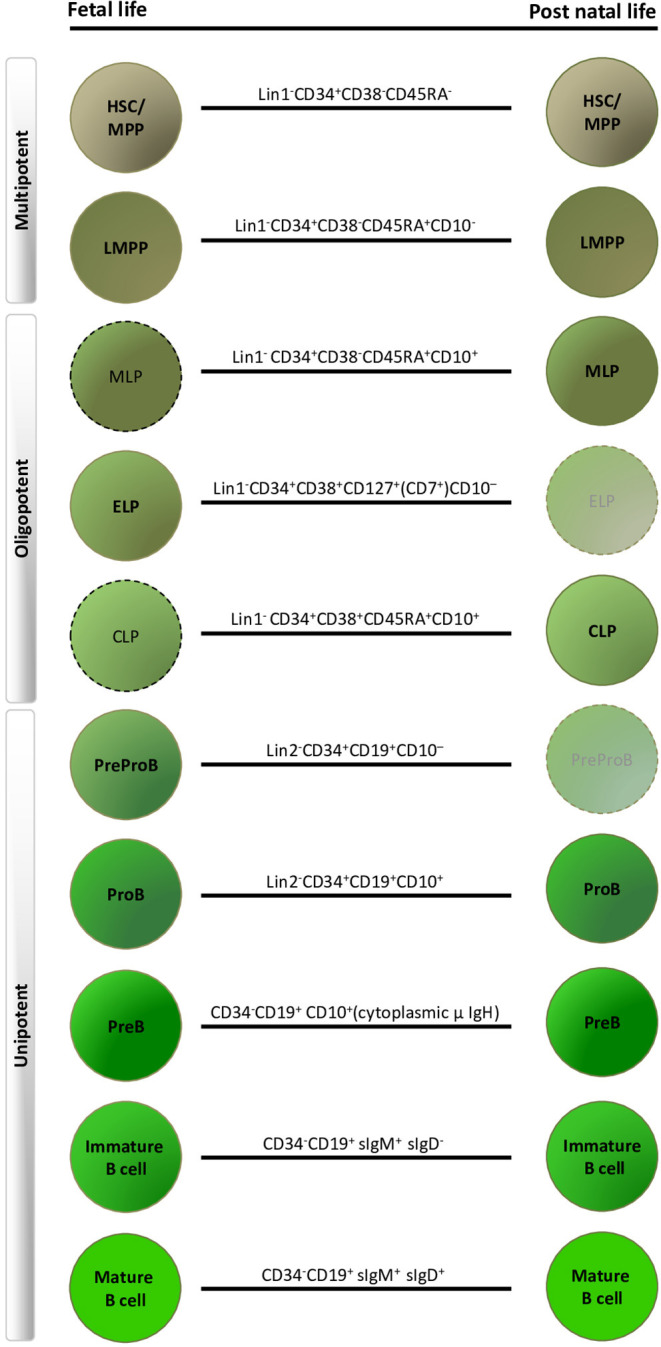
Human fetal and postnatal B-lymphoid compartments and their immunophenotypes. Dashed outlines indicate cell types that have been described immunophenotypically, but have not yet been characterized by detailed functional and molecular profiling. Faded progenitors, postnatal early lymphoid progenitor (ELP) and PreProB-progenitors are exceedingly rare in adult life. HSC, hematopoietic stem cell; MPP, multi-potent progenitors; LMPP, lymphoid-primed multi-potent progenitors; MLP, multi-lymphoid progenitors; CLP, common lymphoid progenitor. Lin1, lineage cocktail 1: CD2/3/14/19/56/235; Lin2, lineage cocktail 2: CD2/3/14/56/235.

The traditional human B-lymphoid developmental hierarchy in adult life demonstrates the following lineage progression in ABM: HSC, multi-potent progenitors (MPP), lymphoid-primed multi-potent progenitors (LMPP) (25, 26), multi-lymphoid progenitors (MLP) (27, 28), common lymphoid progenitors (CLP) (29), ProB-progenitors, PreB-cells and finally mature B-cells (30–32) (Figure 1). Lineage commitment is a multi-stage process defined by transcription factors and their related gene regulatory networks, influenced both by cell intrinsic factors and extracellular signals from the microenvironment (29, 33–35). CD19 expression is the hallmark of B-lineage commitment, with ProB-progenitors being the first CD19^+^ cells in ABM that also initiate immunoglobulin heavy chain V_H_-D_H_-J_H_ rearrangement (31, 36). In recent years, single cell approaches have been extensively applied to delineate cellular hierarchies and molecular pathways in hematopoiesis (37, 38). However, the majority of studies have been done in human cord blood (5, 39) or adult tissues (38, 40, 41).

Recent studies have begun to leverage sophisticated transcriptomic and functional assays to identify B-lymphoid progenitor compartments in the fetus that are not represented in the adult. These, and/or their microenvironment, are hypothesized to be important for the pathogenesis of infant and childhood leukemias, and perhaps also adult malignancies with *in utero* origins (15, 42).

## Human Fetal B-Lymphopoiesis

The timings and sites of fetal hematopoiesis have been broadly mapped out in humans. Hematopoiesis is initiated at day 18 post conception in the yolk sac, independently definitive HSC emerge from the aorta-gonad-mesonephros (AGM) at 4 pcw and subsequently migrate to the FL and then bone marrow, which remains the main site of hematopoiesis after birth (43–47). HSCs colonize the FL from 5th pcw, and they are detectable later in the long bones at 10–12 pcw (1, 48).

In humans, the first evidence of onset of embryonic lymphopoiesis is in the FL at 6 pcw, with multi-potent progenitors (HSC, MPP, LMPP) and fetal-specific oligo-potent early lymphoid progenitors (ELP) detectable. B-progenitors and B-cells are seen in FL by 7 pcw (9, 30, 49, 50). From 2nd trimester the FBM takes over from the FL as the main site of B-lymphopoiesis (1, 51).

### Fetal Lymphoid Progenitors

Interestingly, in murine models immune restricted cells with lymphoid potential are observed in the yolk sac (YS), preceding the first HSCs found in FL; these have potential to produce lymphocytes and granulocyte macrophage progenitors (52) and express Il7 receptor (Il7-r/CD127). Transcriptomic data suggests that such lymphoid progenitors may also be present in human YS (9) but these have not been systematically characterized yet. In humans a potentially analogous cell has been identified in the FL, from 6 pcw; (CD34^+^CD19^−^IL7R^+^) (1, 50, 53, 54). Similar IL7R^+^ progenitors have been described in human FBM (1). FL and FBM CD34^+^CD127^+^CD19^−^CD10^−^ ELP have been characterized by functional and transcriptomic assays, and shown to generate B, T and NK cells while retaining some residual myeloid output. These fetal-specific ELP are very rare in postnatal life (1, 54). There has therefore, been considerable interest in these cells as potential target cells for childhood-ALL.

### Fetal B-Progenitors

From 7 pcw the presence of two committed CD19^+^ B-progenitors downstream of ELP has been confirmed in human FL samples; PreProB (CD34^+^CD19^+^CD10^−^) and ProB (CD34^+^CD19^+^CD10^+^) progenitors; differing in their CD10 expression (1, 50, 54). Similar progenitors have been described in cord blood (55, 56). PreProB-progenitors account for ~2.5% and ProB-progenitors ~8% of FL CD34^+^ cells, and these frequencies remain fairly stable in FL between 7 and 20 pcw. These cells have also been identified by single cell transcriptomic approaches in the human FL (9).

PreProB and ProB-progenitors are also present and markedly expanded in human FBM (1). Both B-progenitor compartments undergo marked expansion in the early stages of colonization of FBM, to account for up to around 20% and 11% of FBM CD34^+^ cells, respectively, at 11 pcw. Later in the second trimester PreProB-progenitors plateau while ProB-progenitors expand further to >30% of CD34^+^ cells in FBM. By contrast, ABM CD34^+^ compartment was found to have only 0.5% PreProB-progenitors and 14% ProB-progenitors (1).

Both PreProB and ProB-progenitors lie downstream of ELP and generate exclusively B-lymphoid progeny *in vitro* and *in vivo*. Functional and molecular studies have established that FBM PreProB-progenitors lie upstream of ProB-progenitors, and are therefore the earliest B-lymphoid restricted progenitors in the fetal B-cell developmental hierarchy (1).

B cell maturation, defined by B cell receptor diversification, commences in B-lymphoid progenitors in fetal life. Fetal ELP and PreProB-progenitors show partial (D_H_-J_H_) IgH rearrangement (1, 54), whereas the more mature ProB-progenitors demonstrate complete V_H_-D_H_-J_H_ rearrangement (1).

### Fetal B-Cells

CD19^+^ B-cells have been reported in FL and FBM by many groups (30, 48, 49, 57–59), and recently been characterized in greater detail (1, 9, 60, 61). Evidence of B cell maturation is demonstrable in human fetal life, with polyclonal CD19^+^IgM^+^ B-cells (60–63). Although FL and FBM immunoglobulin heavy chain repertoires are equally diversified, FL appears to be the main source of IgM natural immunity during the 2nd trimester, and this correlates with the majority of B-cells in 2nd trimester FBM being CD34^−^CD19^+^CD10^+^IgM/D^−^ PreB-cells with a relative lack of more downstream immature and transitional B-cells (60).

### B1 B-Cells and their Putative Progenitors

B cells can be further divided into B1 B-cells of the innate immune system and “conventional” B2 B-cells of the adaptive immune system. This division is well-established in mice, where sIgM^+^CD11b^+^CD5^+^ B1a B-cells were first identified (64, 65) through the search for, the still elusive, cell of origin of adult human CLL (42, 66). B1b B-cells (sIgM^+^CD11b^+^CD5^−^) were subsequently described (67); both these subtypes are seen predominantly in serous cavities. Further characterization of splenic B1 cells have identified them to be CD5^+/−^CD19^hi^CD1d^mid^CD23^−^CD43^+^IgM^hi^IgD^lo^ (68). Murine B1 B-cell progenitors are found in the yolk sac (69) prior to the emergence of the first definitive HSCs in the FL, which have both B1 and B2 B-cell output (70). The B-cell output skews toward B2 B-cells over ontogeny, with B1 B-cell output being exceedingly rare in ABM (65, 71).

Human B1 B-cells and their upstream progenitors have been proposed as the *in utero* cell of origin for infant and childhood-ALL (72) and as having a role in auto-immune disease (73, 74). In humans, B1 B-cells were described in umbilical cord blood and adult peripheral blood. These cells were CD20^+^CD27^+^CD43^+^CD38^lo/int^ and functioned in line with murine counterparts, including spontaneous IgM secretion, constitutional BCR receptor activity and ability to induce allogeneic T cell proliferation (75). Putative B1 B-cells have also been described in human fetal hematopoiesis, with greatest frequencies in 10 pcw FL, decreasing as FBM is colonized (59). After birth, estimates of B1 B-cell populations range from 1 to 10% circulating B-cells, this frequency falls as age increases (76–78).

The progenitors of B1 B-cells in humans remain elusive and contentious. Two theories posit either a lineage (or layered) model where different subtypes arise from different progenitors or a selection model whereby there is interconversion between B1 and B2 B-cells. In humans, CD27 (one of the cell surface markers of B1 B-cells) expression in ABM ProB-cells coincides with *LIN28B* expression levels similar to that seen in FL. These cells mature preferentially to B1-like B-cells compared to their CD27^−^ counterparts. It is not clear whether this relates to a separate lineage or alternative differentiation potential (79).

In summary, human fetal B-lymphopoiesis starts around 6 pcw in FL, with B-cell production happening simultaneously in FL and FBM from 2nd trimester. Hematopoiesis in the FBM is skewed toward B-lymphopoiesis in 2nd trimester. In addition there are fetal-specific B-lymphoid progenitors (ELP and PreProB-progenitors), B-cells (B1 B-cells) and developmental pathways that are different from human adult life (Figure 1).

## Molecular Profile of Fetal B Cell Progenitors

Recent studies suggest that the ontogenic switch of B1 to B2 B-cells in murine B-cell lineage fate of progenitor cells is determined by a combination of intrinsic fetal gene expression programs (*Lin28b*) (80) and extrinsic FL environmental factors (81). Whole transcriptome profiling of murine fetal and adult B cell progenitors showed distinct differences between B-1 and B-2 B-cells as well as between fetal and adult progenitors (82). Although it is well-accepted that human fetal and adult B-lymphopoiesis differ significantly, very few studies have directly compared the molecular pathways underlying these differences. However, both human adult (35, 41, 83) and fetal (1, 9, 84) RNA-seq data sets across many hematopoietic subpopulations have been produced separately and are publicly available for such analyses.

The advent of single cell sequencing technology has allowed the transcriptome of hematopoietic cells to be investigated in unprecedented detail. Recent single-cell transcriptome profiling of human FL and FBM hematopoiesis has demonstrated the transcriptomic changes that drive differentiation in the fetal B cell hierarchy from HSC to mature B-cells; with upregulation of genes such as *SPIB, SP100* and *CTSS* at HSC/MPP to B-lymphoid transition, followed by gradual upregulation of B-cell specific genes such as *MS4A1, CD79B*, and *DNTT* (1, 9).

Although fetal PreProB-progenitors are functionally identical to ProB-progenitors in being restricted to a B-lineage output; these two progenitor subtypes are molecularly distinct in their gene expression and chromatin accessibility patterns, with many myeloid (*MPO, CSF1R*), T-cell (*CD7, CD244*) and stem cell (*SPINK2, PROM1*) genes being accessible and expressed in PreProB-progenitors (1). In addition, when transcriptomes of iALL blasts are compared with different fetal HSPC populations, they most closely match the two fetal-specific progenitor populations, ELP and PreProB-progenitors (1) implicating these cells as potential targets for leukemic transformation.

Direct comparisons focusing on human B-progenitors showed that although adult and fetal counterparts were functionally similar, they did exhibit ontogeny-related transcriptomic differences at a single cell level, with fetal B-progenitors expressing high levels of genes involved in DNA recombination (*DNTT, RAG1*), as well as myeloid genes and known fetal-specific genes such as *LIN28B* (1, 80).

Previous studies have also shown that B cell receptor (BCR) development differs in fetal life, in particular with respect to V_H_-D_H_-J_H_ joining (85). Fetal BCR have a shorter CDR3 length, and show preferential usage of VH6, DHQ52 and the JH3 and JH4 loci compared to postnatal B-cells (60, 86–89).

## Relevance to Childhood-All

The practical importance of characterizing human fetal B-lymphopoiesis is to understand the origins of childhood B-ALL, many of which are initiated before birth. This has led to the suggestion that fetal specific B1 B-cells and their progenitors could be the target cells for leukemia initiation in many subtypes of childhood leukemia. Gene expression signatures from mice which distinguish B1 and B2 B-cells have been mapped to human orthologs; application of these signatures to human pediatric ALL transcriptomic datasets separates B1 B-cell-like ALL subtypes including *ETV6-RUNX1* ALL, from B2 B-cell-like subtypes such as *BCR-ABL1*, hyperdiploid, and *KMT2A* ALL subtypes (90). Intriguingly, in murine models BCR-ABL transduction into B1 B-progenitors yields greater tumor burden in resulting murine leukemia than B2 B-progenitors (91).

These data suggest that it is likely that the biology of different types of infant/childhood Precursor B-ALL depends on the developmental stage specific characteristics of the leukemia-initiating cell although this remains to be demonstrated directly. Nevertheless, it is likely that this is particularly relevant for iALL, which invariably originates *in utero* and presents as a rapid onset aggressive leukemia within the 1st year of life.

### Clinical and Biological Features of Infant and Childhood-ALL

The clinical course and molecular features of iALL are distinct from childhood-ALL. iALL remains a disease with dismal event-free survival (EFS) (18, 92–94), although recent risk-stratified treatment protocols suggest that outcomes could be improved (95). In iALL, blasts are predominantly CD19^+^CD10^−^, often with aberrant myeloid cell surface markers suggestive of an immature B-progenitor, as opposed to a CD19^+^CD10^+^ Pre-B phenotype in childhood-ALL (18, 96). *KMT2A* gene rearrangements (*KMT2A-r*) is the main genetic driver for 70–80% iALL cases, as opposed to only 2–5% of childhood-ALL cases (97, 98).

Current evidence suggests that iALL (particularly *KMT2A-r* ALL) originates *in utero* and has been traced back to its fetal origin through retrospective detection of the fusion gene in neonatal blood spots (99), as well as studies in monozygotic twins with ALL (100, 101). A characteristic feature of iALL is the fact that a single hit (*KMT2A-r*) before birth seems to be sufficient to induce a rapidly-proliferating, therapy-resistant leukemia without the need for additional mutations (102).

**Figure 2:**
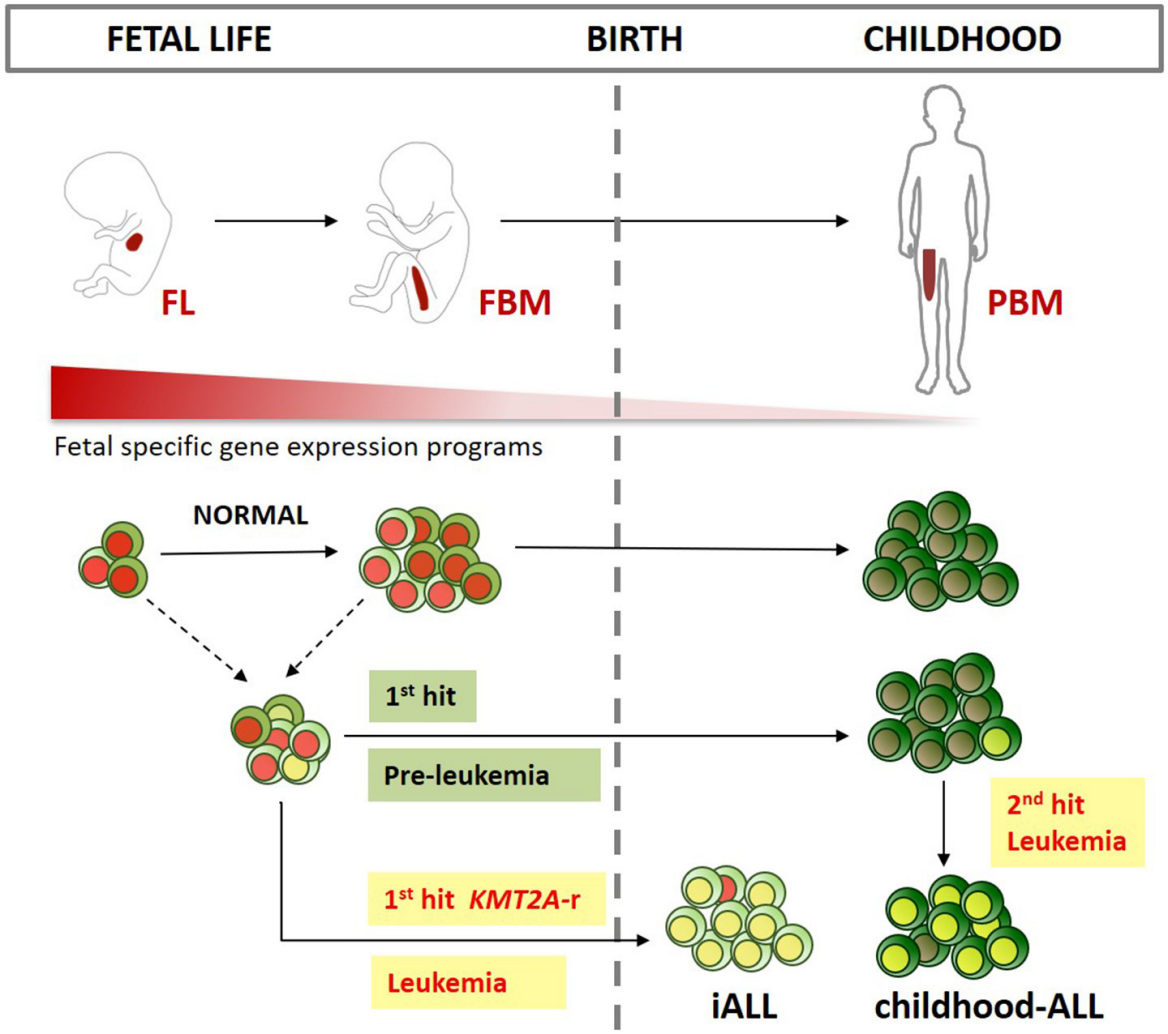
Developmental origins of ALL. Schematic representation of the main sites of early life B-lymphopoiesis (FL, fetal liver; FBM, fetal bone marrow; PBM, pediatric BM). Different fetal lymphoid progenitors could be the target cell for infant ALL (iALL), and childhood-ALL. While iALL can develop after just one intrauterine “hit” such as *KMT2A* gene rearrangement (*KMT2A-r*), childhood-ALL usually develops after a second postnatal hit. Fetal-specific gene expression programs are down regulated after birth. These programs might be key in providing a permissive cellular context for prenatal B-progenitor leukemia initiation in specific target cells as described above.

Unlike iALL, many cases of childhood-ALL also originate *in utero* but only develop into full-blown leukemia after a second post-natal hit (15, 16). Several subtypes of childhood B-ALL have been shown to arise *in utero* including those characterized by *KMT2A-r* (103, 104), *ETV6-RUNX1* (105–107)*, BCR-ABL* (108)*, TCF3-PBX1* (109)*, TCF3-ZNF384* (110) gene fusions and high hyperdiploid ALL (111, 112) (Figure 2).

There are several properties of fetal hematopoietic cells that may underlie the pathogenesis of iALL and childhood-ALL. Firstly, fetal HSPC are more proliferative (6, 7) and have better long term repopulating ability in xenograft models (8, 113–115). Fetal-specific gene expression programs such as the *LIN28B-LET-7-HMGA2* axis (79, 80, 116, 117) have been shown to drive self-renewal (118) and oncogenesis (10–12, 119). Activation of *LIN28B*, in particular, has been demonstrated in several cancers and results in suppression of *LET-7* micro-RNAs and subsequent de-repression of an array of oncogenes including *MYC, RAS, BLIMP1, ARID3A* and *HMGA2* (10, 120). *ARID3A* is necessary for fetal B lymphopoiesis and B1 cell division (121, 122), and has also been shown to promote cancers by driving higher *MYC* expression (123, 124). *HMGA2* is a fetal-specific transcription factor that is re-expressed in many cancers. It promotes cell proliferation, and the Lin28-Let-7-HMGA2 axis maintains cancers in an undifferentiated state (125). The expression of oncogenes such as *LIN28B* in fetal HSPC, may therefore play a role in leukemia initiation and transformation of fetal target cells, and in particular the development of aggressive leukemias in infancy and early childhood.

Secondly, there is a higher proportion of B-progenitors in fetal life compared to adults (1, 2). B-lymphopoiesis itself changes through the human lifetime with a switch in the ratio of B-progenitors to more mature B-cells (30, 49). Regardless of the mechanism, hematopoiesis in the human FBM is skewed toward the B-lymphoid lineage with the presence of a very high frequency of B-progenitors (1, 126) thus expanding the pool of target cells for malignant transformation.

### Developmental Origins of iALL

It is also possible that the fetal cell of origin for iALL and childhood-ALL are different (Figure 2). We suggest that an attractive hypothesis is that iALL arises in a unique B-progenitor found only in fetal life. Of particular interest are fetal-specific IL7R^+^ ELP (1, 50, 53, 54) and PreProB-progenitors (1, 50, 55, 56) that share immunophenotypic, transcriptomic and *IgH* rearrangement patterns with iALL blasts (1, 96). Compared to ABM counterparts, fetal PreProB-progenitors uniquely express known oncofetal genes, such as *LIN28B*, as well as genes implicated in *KMT2A-r* iALL, such as *KLRK1* and *PPP1R14A* (127, 128) that have not previously been recognized as being fetal-specific (1). Fetal ELP/PreProB-progenitors also demonstrate features that could account for lineage plasticity such as an accessible chromatin pattern, together with residual expression, of myeloid and stem cell genes (1). In addition, iALL can switch to a myeloid lineage at relapse, especially after B-lymphoid directed treatment (129–132). This could either be a feature of residual preleukemic primitive progenitors that are capable of giving rise to both myeloid and lymphoid leukemia, or because of plasticity and/or reprogramming of leukemic early B-lymphoid progenitors (130, 133). For example, *KMT2A-r*, the most frequent genetic driver of iALL, may drive leukemogenesis by binding to accessible genes in permissive fetal progenitors; or indeed alter the chromatin accessibility and gene expression patterns of target genes. *KMT2A* is a lysine methyltransferase, and *KMT2A-r* is thought to promote leukemogenesis by activating key target genes such as *HOXA9* and *MEIS1* (134, 135). Although there is some heterogeneity in *KMT2A-r* ALL based on the specific fusion partner gene, most KMT2A-fusion proteins drive and maintain leukemia via a protein complex involving AF4/ENL/AF9/PTEF-B. KMT2A-fusion proteins bind directly to gene targets where they aberrantly upregulate gene expression, partly by increasing histone-3-lysine-79 dimethylation through DOT1L (135).

These mechanisms of *KMT2A-r* mediated transformation are difficult to validate without a *bona fide* model of iALL, which has been very difficult to generate. However, we have recently developed a novel iALL model derived by CRISPR-Cas9 mediated *KMT2A-r* in primary human FL HSPC (136). This demonstrates that a human fetal cell context is permissive, and indeed probably required; to give rise to an ALL that recapitulates key features of iALL. In this model, recruitment of fetal-specific genes by KMT2A-AF4 is demonstrated by KMT2A-N and AF4-C binding and H3K79me2 at these genes by ChIP-seq (136). Furthermore, maintenance of fetal-specific gene expression programs accounts for the unique molecular profile of iALL, suggesting that it is the specific fetal target cell(s) in which it arises that provide the permissive cellular context (136).

### Developmental Origins of Childhood-ALL

It is possible that childhood-ALL on the other hand is likely to arise from a more mature CD19^+^CD10^+^ fetal B-progenitor such as ProB-progenitors or PreB-cells. These cell populations are found in abundance in FBM and expand rapidly throughout the second trimester. As in iALL, several genes that have been implicated in the pathogenesis of childhood-ALL are also important in fetal B lymphoid development. Some of these, such as *PAX5, EBF1, TCF3*, and *IL7R* (137, 138), are expressed at higher levels in fetal B-progenitors compared to postnatal counterparts (1). This is also true for the B-cell specific gene *RAG1* that may play a role in driving childhood-ALL-associated chromosomal translocations such as *ETV6-RUNX1* (139). In addition, childhood-ALL is characterized by multiple lesions affecting cell cycle and B-cell differentiation genes (138). It is hypothesized that the proliferative capacity and complementary epigenetic profile (such as greater chromatin accessibility of highly expressed genes) of the cell of origin provide the right substrate for leukemic transformation (35, 140). This permissive cell-state is likely to be present in FBM ProB-progenitors where their rapid proliferation at the expense of differentiation during a particular developmental time window may make them more susceptible to oncogenic hits. Others have hypothesized that it is the fetal/neonatal BM niche that drives the lymphoid-biased phenotype of *KMT2A-r* infant/childhood leukemia (141).

## Conclusion

Recent advances in developmental hematopoiesis have allowed better characterization of human fetal B-lymphopoiesis using molecular and functional studies. This has revealed fetal-specific B-lymphoid progenitors and B-cell developmental pathways that can be distinguished from postnatal B-lymphopoiesis. Lineage specification of fetal progenitors, the enrichment of multi/oligopotent progenitors and their proliferative capacity is also likely to be driven by microenvironmental cues from the FL and FBM hematopoietic niche.

Studies directly comparing fetal B-lymphoid cells and their microenvironment with childhood and adult counterparts are crucial if we are to understand the site- and stage-specific variation in hematopoiesis throughout the human lifetime and the role it plays in normal and abnormal B-lymphopoiesis. This also has implications for using age-appropriate controls for studies of disorders of hematopoiesis, particularly in early life.

The lymphoid bias of normal fetal hematopoiesis may well be a key factor in the predominance of ALL among infants and children. A better understanding of the importance of the fetal context for leukemogenesis is likely to require models derived from human fetal HSPCs and/or niche. Using human fetal cells to develop faithful infant and childhood-ALL models will allow better understanding of disease pathogenesis and rational development and testing of therapeutics in the future.

## Author Contributions

TJ, RL, and AR drafted the manuscript. AR reviewed and edited the manuscript. All authors read and approved the final manuscript.

## Conflict of Interest

The authors declare that the research was conducted in the absence of any commercial or financial relationships that could be construed as a potential conflict of interest.

## References

[1] O'Byrne S Elliott N Rice S Buck G Fordham N Garnett C. Discovery of a CD10-negative B-progenitor in human fetal life identifies unique ontogeny-related developmental programs. Blood. (2019) 134:1059–71. 10.1182/blood.2019001289 31383639

[2] Rossi MI Yokota T Medina KL Garrett KP Comp PC Schipul AH Jr. B lymphopoiesis is active throughout human life, but there are developmental age-related changes. Blood. (2003) 101:576–84. 10.1182/blood-2002-03-0896 12393702

[3] Pang WW Price EA Sahoo D Beerman I Maloney WJ Rossi DJ. Human bone marrow hematopoietic stem cells are increased in frequency and myeloid-biased with age. Proc Natl Acad Sci. (2011) 108:20012. 10.1073/pnas.1116110108 22123971 PMC3250139

[4] Rundberg Nilsson A Soneji S Adolfsson S Bryder D Pronk CJ. Human and murine hematopoietic stem cell aging is associated with functional impairments and intrinsic megakaryocytic/erythroid bias. PLoS ONE. (2016) 11:e0158369. 10.1371/journal.pone.0158369 27368054 PMC4930192

[5] Notta F Zandi S Takayama N Dobson S Gan OI Wilson G. Distinct routes of lineage development reshape the human blood hierarchy across ontogeny. Science. (2016) 351:aab2116-aab. 10.1126/science.aab2116 26541609 PMC4816201

[6] Lansdorp PM Dragowska W Mayani H. Ontogeny-related changes in proliferative potential of human hematopoietic cells. J Exp Med. (1993) 178:787–91. 10.1084/jem.178.3.787 7688789 PMC2191172

[7] Muench MO Cupp J Polakoff J Roncarolo MG. Expression of CD33, CD38, and HLA-DR on CD34+ human fetal liver progenitors with a high proliferative potential. Blood. (1994) 83:3170–81. 10.1182/blood.V83.11.3170.bloodjournal83113170 7514903

[8] Harrison DE Zhong RK Jordan CT Lemischka IR Astle CM. Relative to adult marrow, fetal liver repopulates nearly five times more effectively long-term than short-term. Exp Hematol. (1997) 25:293–7. 9131003

[9] Popescu D-M Botting RA Stephenson E Green K Webb S Jardine L. Decoding human fetal liver haematopoiesis. Nature. (2019) 574:365–71. 10.1038/s41586-019-1652-y 31597962 PMC6861135

[10] Zhou J Ng S-B Chng W-J. LIN28/LIN28B: an emerging oncogenic driver in cancer stem cells. Int J Biochem Cell Biol. (2013) 45:973–8. 10.1016/j.biocel.2013.02.006 23420006

[11] Viswanathan SR Powers JT Einhorn W Hoshida Y Ng TL Toffanin S. Lin28 promotes transformation and is associated with advanced human malignancies. Nat Genet. (2009) 41:843–8. 10.1038/ng.392 19483683 PMC2757943

[12] Elcheva IA Wood T Chiarolanzio K Chim B Wong M Singh V. RNA-binding protein IGF2BP1 maintains leukemia stem cell properties by regulating HOXB4, MYB, and ALDH1A1. Leukemia. (2020) 34:1354–63. 10.1038/s41375-019-0656-9 31768017 PMC7196026

[13] Kuijk E Blokzijl F Jager M Besselink N Boymans S Chuva De Sousa Lopes SM. Early divergence of mutational processes in human fetal tissues. Sci Adv. (2019) 5:eaaw1271. 10.1126/sciadv.aaw1271 31149636 PMC6541467

[14] Hasaart KAL Manders F van der Hoorn M-L Verheul M Poplonski T Kuijk E. Mutation accumulation and developmental lineages in normal and Down syndrome human fetal haematopoiesis. Sci Rep. (2020) 10:12991. 10.1038/s41598-020-69822-1 32737409 PMC7395765

[15] Greaves M. *In utero* origins of childhood leukaemia. Early Hum Dev. (2005) 81:123–9. 10.1016/j.earlhumdev.2004.10.004 15707724

[16] Greaves M. A causal mechanism for childhood acute lymphoblastic leukaemia. Nat Rev Cancer. (2018) 18:471–84. 10.1038/s41568-018-0015-6 29784935 PMC6986894

[17] Vora A Goulden N Wade R Mitchell C Hancock J Hough R. Treatment reduction for children and young adults with low-risk acute lymphoblastic leukaemia defined by minimal residual disease (UKALL 2003): a randomised controlled trial. Lancet Oncol. (2013) 14:199–209. 10.1016/S1470-2045(12)70600-9 23395119

[18] Pieters R De Lorenzo P Ancliffe P Aversa LA Brethon B Biondi A. Outcome of infants younger than 1 year with acute lymphoblastic leukemia treated with the interfant-06 protocol: results from an international phase III randomized study. J Clin Oncol. (2019) 37:2246. 10.1200/JCO.19.00261 31283407

[19] Doulatov S Notta F Laurenti E John. Hematopoiesis: a human perspective. Cell Stem Cell. (2012) 10:120–36. 10.1016/j.stem.2012.01.006 22305562

[20] Ernst PB Carvunis AR. Of mice, men and immunity: a case for evolutionary systems biology. Nat Immunol. (2018) 19:421–5. 10.1038/s41590-018-0084-4 29670240 PMC6168288

[21] Milne TA. Mouse models of MLL leukemia: recapitulating the human disease. Blood. (2017) 129:2217–23. 10.1182/blood-2016-10-691428 28179274 PMC5399479

[22] Rice S Roy A. MLL-rearranged infant leukaemia: a 'thorn in the side' of a remarkable success story. Biochim Biophys Acta Gene Regul Mech. (2020) 1863:194564. 10.1016/j.bbagrm.2020.194564 32376390

[23] Höfer T Rodewald H-R. Differentiation-based model of hematopoietic stem cell functions and lineage pathways. Blood. (2018) 132:1106–13. 10.1182/blood-2018-03-791517 30042097 PMC6307983

[24] Eaves CJ. Hematopoietic stem cells: concepts, definitions, and the new reality. Blood. (2015) 125:2605–13. 10.1182/blood-2014-12-570200 25762175 PMC4440889

[25] Kohn LA Hao Q-L Sasidharan R Parekh C Ge S Zhu Y. Lymphoid priming in human bone marrow begins before expression of CD10 with upregulation of L-selectin. Nat Immunol. (2012) 13:963–71. 10.1038/ni.2405 22941246 PMC3448017

[26] Luc S Buza-Vidas N Jacobsen SEW. Biological and molecular evidence for existence of lymphoid-primed multipotent progenitors. Ann N Y Acad Sci. (2007) 1106:89–94. 10.1196/annals.1392.023 17442777

[27] Doulatov S Notta F Eppert K Nguyen LT Ohashi PS Dick JE. Revised map of the human progenitor hierarchy shows the origin of macrophages and dendritic cells in early lymphoid development. Nat Immunol. (2010) 11:585–93. 10.1038/ni.1889 20543838

[28] Karamitros D Stoilova B Aboukhalil Z Hamey F Reinisch A Samitsch M. Single-cell analysis reveals the continuum of human lympho-myeloid progenitor cells. Nat Immunol. (2018) 19:85–97. 10.1038/s41590-017-0001-2 29167569 PMC5884424

[29] Galy A Travis M Cen D Chen B. Human T, B, natural killer, and dendritic cells arise from a common bone marrow progenitor cell subset. Immunity. (1995) 3:459–73. 10.1016/1074-7613(95)90175-2 7584137

[30] Nuñez C Nishimoto N Gartland GL Billips LG Burrows PD Kubagawa H. B cells are generated throughout life in humans. J Immunol. (1996) 156:866–72. 8543844

[31] LeBien TW. Fates of human B-cell precursors. Blood. (2000) 96:9–23. 10.1182/blood.V96.1.9.013k27_9_23 10891425

[32] Hystad ME Myklebust JH Bo TH Sivertsen EA Rian E Forfang L. Characterization of early stages of human B cell development by gene expression profiling. J Immunol. (2007) 179:3662–71. 10.4049/jimmunol.179.6.3662 17785802

[33] Boller S Grosschedl R. The regulatory network of B-cell differentiation: a focused view of early B-cell factor 1 function. Immunol Rev. (2014) 261:102–15. 10.1111/imr.12206 25123279 PMC4312928

[34] Blom B Spits H. Development of human lymphoid cells. Annu Rev Immunol. (2006) 24:287–320. 10.1146/annurev.immunol.24.021605.090612 16551251

[35] Corces MR Buenrostro JD Wu B Greenside PG Chan SM Koenig JL. Lineage-specific and single-cell chromatin accessibility charts human hematopoiesis and leukemia evolution. Nat Genet. (2016) 48:1193–203. 10.1038/ng.3646 27526324 PMC5042844

[36] Van Zelm MC Van Der Burg M De Ridder D Barendregt BH De Haas EFE Reinders MJT. Ig gene rearrangement steps are initiated in early human precursor B cell subsets and correlate with specific transcription factor expression. J Immunol. (2005) 175:5912–22. 10.4049/jimmunol.175.9.5912 16237084

[37] Laurenti E Göttgens B. From haematopoietic stem cells to complex differentiation landscapes. Nature. (2018) 553:418–26. 10.1038/nature25022 29364285 PMC6555401

[38] Velten L Haas SF Raffel S Blaszkiewicz S Islam S Hennig BP. Human haematopoietic stem cell lineage commitment is a continuous process. Nat Cell Biol. (2017) 19:271–81. 10.1038/ncb3493 28319093 PMC5496982

[39] Zheng S Papalexi E Butler A Stephenson W Satija R. Molecular transitions in early progenitors during human cord blood hematopoiesis. Mol Syst Biol. (2018) 14:e8041-e. 10.15252/msb.20178041 29545397 PMC5852373

[40] Bendall SC Davis KL Amir el AD Tadmor MD Simonds EF Chen TJ. Single-cell trajectory detection uncovers progression and regulatory coordination in human B cell development. Cell. (2014) 157:714–25. 10.1016/j.cell.2014.04.005 24766814 PMC4045247

[41] Hay SB Ferchen K Chetal K Grimes HL Salomonis N. The Human Cell Atlas bone marrow single-cell interactive web portal. Exp Hematol. (2018) 68:51–61. 10.1016/j.exphem.2018.09.004 30243574 PMC6296228

[42] Hardy RR Hayakawa K. Perspectives on fetal derived CD5+B1 B cells. Eur J Immunol. (2015) 45:2978–84. 10.1002/eji.201445146 26339791 PMC4674377

[43] Tavian M Hallais MF Peault B. Emergence of intraembryonic hematopoietic precursors in the pre-liver human embryo. Development. (1999) 126:793–803. 9895326 10.1242/dev.126.4.793

[44] Tavian M Coulombel L Luton D Clemente HS Dieterlen-Lievre F Peault B. Aorta-associated CD34+ hematopoietic cells in the early human embryo. Blood. (1996) 87:67–72. 10.1182/blood.V87.1.67.67 8547678

[45] Tavian M Peault B. Embryonic development of the human hematopoietic system. Int J Dev Biol. (2005) 49:243–50. 10.1387/ijdb.041957mt 15906238

[46] Copley MR Eaves CJ. Developmental changes in hematopoietic stem cell properties. Exp Mol Med. (2013) 45:e55. 10.1038/emm.2013.98 24232254 PMC3849580

[47] Ivanovs A Rybtsov S Ng ES Stanley EG Elefanty AG Medvinsky A. Human haematopoietic stem cell development: from the embryo to the dish. Development. (2017) 144:2323–37. 10.1242/dev.134866 28676567

[48] Charbord P Tavian M Humeau L Peault B. Early ontogeny of the human marrow from long bones: an immunohistochemical study of hematopoiesis and its microenvironment. Blood. (1996) 87:4109–19. 10.1182/blood.V87.10.4109.bloodjournal87104109 8639768

[49] Asma GE Langlois van den Bergh R Vossen JM. Development of pre-B and B lymphocytes in the human fetus. Clin Exp Immunol. (1984) 56:407–14. 6610515 PMC1536228

[50] Roy A Cowan G Mead AJ Filippi S Bohn G Chaidos A. Perturbation of fetal liver hematopoietic stem and progenitor cell development by trisomy 21. Proc Natl Acad Sci. (2012) 109:17579–84. 10.1073/pnas.1211405109 23045701 PMC3491522

[51] Tavian M Biasch K Sinka L Vallet J Peault B. Embryonic origin of human hematopoiesis. Int J Dev Biol. (2010) 54:1061–5. 10.1387/ijdb.103097mt 20711983

[52] Böiers C Carrelha J Lutteropp M Luc S Joanna Azzoni E. Lymphomyeloid contribution of an immune-restricted progenitor emerging prior to definitive hematopoietic stem cells. Cell Stem Cell. (2013) 13:535–48. 10.1016/j.stem.2013.08.012 24054998

[53] Alhaj Hussen K Vu Manh TP Guimiot F Nelson E Chabaane E Delord M. Molecular and functional characterization of lymphoid progenitor subsets reveals a bipartite architecture of human lymphopoiesis. Immunity. (2017) 47:680–96 e8. 10.1016/j.immuni.2017.09.009 29045900

[54] Böiers C Richardson SE Laycock E Zriwil A Turati VA Brown J. A human IPS model implicates embryonic B-myeloid fate restriction as developmental susceptibility to B Acute lymphoblastic leukemia-associated ETV6-RUNX1. Dev Cell. (2018) 44:362–77.e7. 10.1016/j.devcel.2017.12.005 29290585 PMC5807056

[55] Sanz E Alvarez-Mon M Martinez AC de la Hera A. Human cord blood CD34+Pax-5+ B-cell progenitors: single-cell analyses of their gene expression profiles. Blood. (2003) 101:3424–30. 10.1182/blood-2002-07-2244 12446447

[56] Sanz E Munoz AN Monserrat J Van-Den-Rym A Escoll P Ranz I. Ordering human CD34+CD10-CD19+ pre/pro-B-cell and CD19- common lymphoid progenitor stages in two pro-B-cell development pathways. Proc Natl Acad Sci U S A. (2010) 107:5925–30. 10.1073/pnas.0907942107 20231472 PMC2851857

[57] Grumayer ER Griesinger F Hummell DS Brunning RD Kersey JH. Identification of novel B-lineage cells in human fetal bone marrow that coexpress CD7. Blood. (1991) 77:64–8. 10.1182/blood.V77.1.64.64 1702030

[58] Uckun FM Ledbetter JA. Immunobiologic differences between normal and leukemic human B-cell precursors. Proc Natl Acad Sci U S A. (1988) 85:8603–7. 10.1073/pnas.85.22.8603 2460871 PMC282507

[59] Bueno C Van Roon EHJ Muñoz-López A Sanjuan-Pla A Juan M Navarro A. Immunophenotypic analysis and quantification of B-1 and B-2 B cells during human fetal hematopoietic development. Leukemia. (2016) 30:1603–6. 10.1038/leu.2015.362 26710885

[60] Roy A Bystry V Bohn G Goudevenou K Reigl T Papaioannou M. High resolution IgH repertoire analysis reveals fetal liver as the likely origin of life-long, innate B lymphopoiesis in humans. Clin Immunol. (2017) 183:8–16. 10.1016/j.clim.2017.06.005 28645875 PMC5678457

[61] Rechavi E Lev A Lee YN Simon AJ Yinon Y Lipitz S. Timely and spatially regulated maturation of B and T cell repertoire during human fetal development. Sci Transl Med. (2015) 7:276ra25. 10.1126/scitranslmed.aaa0072 25717098

[62] Berman JE Nickerson KG Pollock RR Barth JE Schuurman RK Knowles DM. VH gene usage in humans: biased usage of the VH6 gene in immature B lymphoid cells. Eur J Immunol. (1991) 21:1311–4. 10.1002/eji.1830210532 1903708

[63] Pascual V Verkruyse L Casey ML Capra JD. Analysis of Ig H chain gene segment utilization in human fetal liver. Revisiting the “proximal utilization hypothesis”. J Immunol. (1993) 151:4164–72. 8409393

[64] Hayakawa K Hardy RR Parks DR Herzenberg LA. The “Ly-1 B” cell subpopulation in normal immunodefective, and autoimmune mice. J Exp Med. (1983) 157:202–18. 10.1084/jem.157.1.202 6600267 PMC2186909

[65] Hayakawa K Hardy RR Herzenberg LA Herzenberg LA. Progenitors for Ly-1 B cells are distinct from progenitors for other B cells. J Exp Med. (1985) 161:1554–68. 10.1084/jem.161.6.1554 3874257 PMC2187623

[66] Fabbri G Dalla-Favera R. The molecular pathogenesis of chronic lymphocytic leukaemia. Nat Rev Cancer. (2016) 16:145–62. 10.1038/nrc.2016.8 26911189

[67] Kantor AB Herzenberg LA. Origin of murine B cell lineages. Annu Rev Immunol. (1993) 11:501–38. 10.1146/annurev.iy.11.040193.002441 8476571

[68] Baumgarth N. The double life of a B-1 cell: self-reactivity selects for protective effector functions. Annu Rev Immunol. (2011) 11:34–46. 10.1038/nri2901 21151033

[69] Yoshimoto M Montecino-Rodriguez E Ferkowicz MJ Porayette P Shelley WC Conway SJ. Embryonic day 9 yolk sac and intra-embryonic hemogenic endothelium independently generate a B-1 and marginal zone progenitor lacking B-2 potential. Proc Natl Acad Sci U S A. (2011) 108:1468–73. 10.1073/pnas.1015841108 21209332 PMC3029764

[70] Kristiansen TA Vanhee S Yuan J. The influence of developmental timing on B cell diversity. Curr Opin Immunol. (2018) 51:7–13. 10.1016/j.coi.2017.12.005 29272734

[71] Montecino-Rodriguez E Leathers H Dorshkind K. Identification of a B-1 B cell–specified progenitor. Nature Immunology. (2006) 7:293–301. 10.1038/ni1301 16429139

[72] Montecino-Rodriguez E Dorshkind K. B-1 B cell development in the fetus and adult. Immunity. (2012) 36:13–21. 10.1016/j.immuni.2011.11.017 22284417 PMC3269035

[73] Lee J Kuchen S Fischer R Chang S Lipsky PE. Identification and characterization of a human CD5+ pre-naive B cell population. J Immunol. (2009) 182:4116–26. 10.4049/jimmunol.0803391 19299709

[74] Taniguchi O Miyajima H Hirano T Noguchi M Ueda A Hashimoto H. The Leu-1 B-cell subpopulation in patients with rheumatoid arthritis. J Clin Immunol. (1987) 7:441–8. 10.1007/BF00915053 3500961

[75] Griffin DO Holodick NE Rothstein TL. Human B1 cells in umbilical cord and adult peripheral blood express the novel phenotype CD20+CD27+CD43+CD70–. J Exp Med. (2011) 208:67–80. 10.1084/jem.20101499 21220451 PMC3023138

[76] Rodriguez-Zhurbenko N Quach TD Hopkins TJ Rothstein TL Hernandez AM. Human B-1 cells and B-1 cell antibodies change with advancing age. Front Immunol. (2019) 10:483. 10.3389/fimmu.2019.00483 30941130 PMC6433875

[77] Descatoire M Weill J-C Reynaud C-A Weller S. A human equivalent of mouse B-1 cells? J Exp Med. (2011) 208:2563–4. 10.1084/jem.20112232 22184680 PMC3244035

[78] Quách TD Rodríguez-Zhurbenko N Hopkins TJ Guo X Hernández AM Li W. Distinctions among circulating antibody-secreting cell populations, including B-1 cells, in human adult peripheral blood. J Immunol. (2016) 196:1060–9. 10.4049/jimmunol.1501843 26740107 PMC5351554

[79] McWilliams L Su K-Y Liang X Liao D Floyd S Amos J. The human fetal lymphocyte lineage: identification by CD27 and LIN28B expression in B cell progenitors. J Leukoc Biol. (2013) 94:991–1001. 10.1189/jlb.0113048 23901121 PMC3800071

[80] Yuan J Nguyen CK Liu X Kanellopoulou C Muljo SA. Lin28b reprograms adult bone marrow hematopoietic progenitors to mediate fetal-like lymphopoiesis. Science. (2012) 335:1195–200. 10.1126/science.1216557 22345399 PMC3471381

[81] Xu X Deobagkar-Lele M Bull KR Crockford TL Mead AJ Cribbs AP. An ontogenetic switch drives the positive and negative selection of B cells. Proc Natl Acad Sci. (2020) 117:3718–27. 10.1073/pnas.1915247117 32019891 PMC7035474

[82] Montecino-Rodriguez E Fice M Casero D Berent-Maoz B Barber CL Dorshkind K. Distinct genetic networks orchestrate the emergence of specific waves of fetal and adult B-1 and B-2 development. Immunity. (2016) 45:527–39. 10.1016/j.immuni.2016.07.012 27566938 PMC5033716

[83] Quek L Otto GW Garnett C Lhermitte L Karamitros D Stoilova B. Genetically distinct leukemic stem cells in human CD34- acute myeloid leukemia are arrested at a hemopoietic precursor-like stage. J Exp Med. (2016) 213:1513–35. 10.1084/jem.20151775 27377587 PMC4986529

[84] Agraz-Doblas A Bueno C Bashford-Rogers R Roy A Schneider P Bardini M. Unravelling the cellular origin and clinical prognostic markers of infant B-cell acute lymphoblastic leukemia using genome-wide analysis. Haematologica. (2019) 104:1176–88. 10.3324/haematol.2018.206375 30679323 PMC6545849

[85] Schroeder HW Jr Mortari F Shiokawa S Kirkham PM Elgavish RA Bertrand FE III. Developmental regulation of the human antibody repertoire. Ann N Y Acad Sci. (1995) 764:242–60. 10.1111/j.1749-6632.1995.tb55834.x 7486531

[86] Schroeder HW Jr Wang JY. Preferential utilization of conserved immunoglobulin heavy chain variable gene segments during human fetal life. Proc Natl Acad Sci U S A. (1990) 87:6146–50. 10.1073/pnas.87.16.6146 2117273 PMC54489

[87] Souto-Carneiro MM Sims GP Girschik H Lee J Lipsky PE. Developmental changes in the human heavy chain CDR3. J Immunol. (2005) 175:7425–36. 10.4049/jimmunol.175.11.7425 16301650

[88] Van Es JH Raaphorst FM van Tol MJ Meyling FH Logtenberg T. Expression pattern of the most JH-proximal human VH gene segment (VH6) in the B cell and antibody repertoire suggests a role of VH6-encoded IgM antibodies in early ontogeny. J Immunol. (1993) 150:161–8. 8417121

[89] Bertrand FE III Billips LG Burrows PD Gartland GL Kubagawa H Schroeder HW Jr. Ig D(H) gene segment transcription and rearrangement before surface expression of the pan-B-cell marker CD19 in normal human bone marrow. Blood. (1997) 90:736–44. 10.1182/blood.V90.2.736.736_736_744 9226174

[90] Fitch B Roy R Geng H Montecino-Rodriguez E Bengtsson H Gaillard C. Human pediatric B-cell acute lymphoblastic leukemias can be classified as B-1 or B-2-like based on a minimal transcriptional signature. Exp Hematol. (2020) 90:65–71.e1. 10.1016/j.exphem.2020.09.184 32946981 PMC7606616

[91] Montecino-Rodriguez E Li K Fice M Dorshkind K. Murine B-1 B cell progenitors initiate B-acute lymphoblastic leukemia with features of high-risk disease. J Immunol. (2014) 192:5171–8. 10.4049/jimmunol.1303170 24752443 PMC4028370

[92] Dreyer ZE Hilden JM Jones TL Devidas M Winick NJ Willman CL. Intensified chemotherapy without SCT in infant ALL: results from COG P9407 (Cohort 3). Pediatr Blood Cancer. (2015) 62:419–26. 10.1002/pbc.25322 25399948 PMC5145261

[93] Hilden JM Dinndorf PA Meerbaum SO Sather H Villaluna D Heerema NA. Analysis of prognostic factors of acute lymphoblastic leukemia in infants: report on CCG 1953 from the Children's Oncology Group. Blood. (2006) 108:441–51. 10.1182/blood-2005-07-3011 16556894 PMC1895499

[94] Pieters R Schrappe M De Lorenzo P Hann I De Rossi G Felice M. A treatment protocol for infants younger than 1 year with acute lymphoblastic leukaemia (Interfant-99): an observational study and a multicentre randomised trial. Lancet. (2007) 370:240–50. 10.1016/S0140-6736(07)61126-X 17658395

[95] Tomizawa D Miyamura T Imamura T Watanabe T Moriya Saito A Ogawa A. A risk-stratified therapy for infants with acute lymphoblastic leukemia: a report from the JPLSG MLL-10 trial. Blood. (2020) 136:1813–23. 10.1182/blood.2019004741 32845001

[96] Jansen MW Corral L van der Velden VH Panzer-Grumayer R Schrappe M Schrauder A. Immunobiological diversity in infant acute lymphoblastic leukemia is related to the occurrence and type of MLL gene rearrangement. Leukemia. (2007) 21:633–41. 10.1038/sj.leu.2404578 17268512

[97] Iacobucci I Mullighan CG. Genetic basis of acute lymphoblastic leukemia. J Clin Oncol. (2017) 35:975–83. 10.1200/JCO.2016.70.7836 28297628 PMC5455679

[98] Moorman AV Ensor HM Richards SM Chilton L Schwab C Kinsey SE. Prognostic effect of chromosomal abnormalities in childhood B-cell precursor acute lymphoblastic leukaemia: results from the UK Medical Research Council ALL97/99 randomised trial. Lancet Oncol. (2010) 11:429–38. 10.1016/S1470-2045(10)70066-8 20409752

[99] Gale KB Ford AM Repp R Borkhardt A Keller C Eden OB. Backtracking leukemia to birth: identification of clonotypic gene fusion sequences in neonatal blood spots. Proc Natl Acad Sci U S A. (1997) 94:13950–4. 10.1073/pnas.94.25.13950 9391133 PMC28413

[100] Greaves MF Maia AT Wiemels JL Ford AM. Leukemia in twins: lessons in natural history. Blood. (2003) 102:2321–33. 10.1182/blood-2002-12-3817 12791663

[101] Greaves MF Wiemels J. Origins of chromosome translocations in childhood leukaemia. Nat Rev Cancer. (2003) 3:639–49. 10.1038/nrc1164 12951583

[102] Andersson AK Ma J Wang J Chen X Gedman AL Dang J. The landscape of somatic mutations in infant MLL-rearranged acute lymphoblastic leukemias. Nat Genet. (2015) 47:330–7. 10.1038/ng.3230 25730765 PMC4553269

[103] Ford AM Ridge SA Cabrera ME Mahmoud H Steel CM Chan LC. *In utero* rearrangements in the trithorax-related oncogene in infant leukaemias. Nature. (1993) 363:358–60. 10.1038/363358a0 8497319

[104] Jones LK Neat MJ van Delft FW Mitchell MP Adamaki M Stoneham SJ. Cryptic rearrangement involving MLL and AF10 occurring *in utero*. Leukemia. (2003) 17:1667–9. 10.1038/sj.leu.2403039 12886258

[105] Wiemels JL Ford AM Van Wering ER Postma A Greaves M. Protracted and variable latency of acute lymphoblastic leukemia after TEL-AML1 gene fusion *in utero*. Blood. (1999) 94:1057–62. 10.1182/blood.V94.3.1057.415k10_1057_1062 10419898

[106] Ford AM Bennett CA Price CM Bruin MC Van Wering ER Greaves M. Fetal origins of the TEL-AML1 fusion gene in identical twins with leukemia. Proc Natl Acad Sci U S A. (1998) 95:4584–8. 10.1073/pnas.95.8.4584 9539781 PMC22533

[107] Hong D Gupta R Ancliff P Atzberger A Brown J Soneji S. Initiating and cancer-propagating cells in TEL-AML1-associated childhood leukemia. Science. (2008) 319:336–9. 10.1126/science.1150648 18202291

[108] Cazzaniga G van Delft FW Lo Nigro L Ford AM Score J Iacobucci I. Developmental origins and impact of BCR-ABL1 fusion and IKZF1 deletions in monozygotic twins with Ph+ acute lymphoblastic leukemia. Blood. (2011) 118:5559–64. 10.1182/blood-2011-07-366542 21960589 PMC3217357

[109] Hein D Dreisig K Metzler M Izraeli S Schmiegelow K Borkhardt A. The preleukemic TCF3-PBX1 gene fusion can be generated in utero and is present in ≈0.6% of healthy newborns. Blood. (2019) 134:1355–8. 10.1182/blood.2019002215 31434706 PMC7005361

[110] Bueno C Tejedor JR Bashford-Rogers R González-Silva L Valdés-Mas R Agraz-Doblás A. Natural history and cell of origin of TCF3-ZNF384 and PTPN11 mutations in monozygotic twins with concordant BCP-ALL. Blood. (2019) 134:900–5. 10.1182/blood.2019000893 31221673

[111] Panzer-Grümayer ER Fasching K Panzer S Hettinger K Schmitt K Stöckler-Ipsiroglu S. Nondisjunction of chromosomes leading to hyperdiploid childhood B-cell precursor acute lymphoblastic leukemia is an early event during leukemogenesis. Blood. (2002) 100:347–9. 10.1182/blood-2002-01-0144 12070048

[112] Wiemels JL Kang M Chang JS Zheng L Kouyoumji C Zhang L. Backtracking RAS mutations in high hyperdiploid childhood acute lymphoblastic leukemia. Blood Cells Mol Dis. (2010) 45:186–91. 10.1016/j.bcmd.2010.07.007 20688547 PMC2943008

[113] Nicolini FE Holyoake TL Cashman JD Chu PP Lambie K Eaves CJ. Unique differentiation programs of human fetal liver stem cells shown both *in vitro* and *in vivo* in NOD/SCID mice. Blood. (1999) 94:2686–95. 10.1182/blood.V94.8.2686.420k15_2686_2695 10515872

[114] Holyoake TL Nicolini FE Eaves CJ. Functional differences between transplantable human hematopoietic stem cells from fetal liver, cord blood, and adult marrow. Exp Hematol. (1999) 27:1418–27. 10.1016/S0301-472X(99)00078-8 10480433

[115] Beyer AI Muench MO. Comparison of human hematopoietic reconstitution in different strains of immunodeficient mice. Stem Cells Dev. (2017) 26:102–12. 10.1089/scd.2016.0083 27758159 PMC5248550

[116] Li YS Zhou Y Tang L Shinton SA Hayakawa K Hardy RR. A developmental switch between fetal and adult B lymphopoiesis. Ann N Y Acad Sci. (2015) 1362:8–15. 10.1111/nyas.12769 25931205

[117] Zhou J Bi C Ching YQ Chooi JY Lu X Quah JY. Inhibition of LIN28B impairs leukemia cell growth and metabolism in acute myeloid leukemia. J Hematol Oncol. (2017) 10:138. 10.1186/s13045-017-0507-y 28693523 PMC5504806

[118] Copley MR Babovic S Benz C Knapp DJ Beer PA Kent DG. The Lin28b-let-7-Hmga2 axis determines the higher self-renewal potential of fetal haematopoietic stem cells. Nat Cell Biol. (2013) 15:916–25. 10.1038/ncb2783 23811688

[119] Oliveira-Mateos C Sanchez-Castillo A Soler M Obiols-Guardia A Pineyro D Boque-Sastre R. The transcribed pseudogene RPSAP52 enhances the oncofetal HMGA2-IGF2BP2-RAS axis through LIN28B-dependent and independent let-7 inhibition. Nat Commun. (2019) 10:3979. 10.1038/s41467-019-11910-6 31484926 PMC6726650

[120] Balzeau J Menezes MR Cao S Hagan JP. The LIN28/let-7 pathway in cancer. Front Genet. (2017) 8:31. 10.3389/fgene.2017.00031 28400788 PMC5368188

[121] Hayakawa K Li YS Shinton SA Bandi SR Formica AM Brill-Dashoff J. Crucial role of increased Arid3a at the Pre-B and immature B cell stages for B1a cell generation. Front Immunol. (2019) 10:457. 10.3389/fimmu.2019.00457 30930899 PMC6428705

[122] Zhou Y Li YS Bandi SR Tang L Shinton SA Hayakawa K. Lin28b promotes fetal B lymphopoiesis through the transcription factor Arid3a. J Exp Med. (2015) 212:569–80. 10.1084/jem.20141510 25753579 PMC4387290

[123] Dausinas P Pulakanti K Rao S Cole JM Dahl R Cowden Dahl KD. ARID3A and ARID3B induce stem promoting pathways in ovarian cancer cells. Gene. (2020) 738:144458. 10.1016/j.gene.2020.144458 32061921 PMC7384259

[124] Tang J Yang L Li Y Ning X Chaulagain A Wang T. ARID3A promotes the development of colorectal cancer by upregulating AURKA. Carcinogenesis. (2020):bgaa118. 10.1093/carcin/bgaa118. [Epub ahead of print]. 33165575

[125] Zhang S Mo Q Wang X. Oncological role of HMGA2 (Review). Int J Oncol. (2019) 55:775–88. 10.3892/ijo.2019.4856 31432151

[126] Roy A PBea. Single-cell profiling reveals key differences in the cellular architecture of human haematopoietic stem and progenitor cells throughout fetal and adult life (23rd Congress of the European Hematology Association Stockholm, Sweden, June 14-17, 2018). HemaSphere. (2018) 2:1–1113. 10.1097/HS9.0000000000000060

[127] Trentin L Giordan M Dingermann T Basso G Te Kronnie G Marschalek R. Two independent gene signatures in pediatric t(4;11) acute lymphoblastic leukemia patients. Eur J Haematol. (2009) 83:406–19. 10.1111/j.1600-0609.2009.01305.x 19558506

[128] Armstrong SA Staunton JE Silverman LB Pieters R Den Boer ML Minden MD. MLL translocations specify a distinct gene expression profile that distinguishes a unique leukemia. Nat Genet. (2002) 30:41–7. 10.1038/ng765 11731795

[129] Gardner R Wu D Cherian S Fang M Hanafi LA Finney O. Acquisition of a CD19-negative myeloid phenotype allows immune escape of MLL-rearranged B-ALL from CD19 CAR-T-cell therapy. Blood. (2016) 127:2406–10. 10.1182/blood-2015-08-665547 26907630 PMC4874221

[130] Jacoby E Nguyen SM Fountaine TJ Welp K Gryder B Qin H. CD19 CAR immune pressure induces B-precursor acute lymphoblastic leukaemia lineage switch exposing inherent leukaemic plasticity. Nat Commun. (2016) 7:12320. 10.1038/ncomms12320 27460500 PMC4974466

[131] Rayes A McMasters RL O'Brien MM. Lineage switch in MLL-rearranged infant leukemia following CD19-directed therapy. Pediatr Blood Cancer. (2016) 63:1113–5. 10.1002/pbc.25953 26914337

[132] Rossi JG Bernasconi AR Alonso CN Rubio PL Gallego MS Carrara CA. Lineage switch in childhood acute leukemia: an unusual event with poor outcome. Am J Hematol. (2012) 87:890–7. 10.1002/ajh.23266 22685031

[133] Wolfl M Rasche M Eyrich M Schmid R Reinhardt D Schlegel PG. Spontaneous reversion of a lineage switch following an initial blinatumomab-induced ALL-to-AML switch in MLL-rearranged infant ALL. Blood Adv. (2018) 2:1382–5. 10.1182/bloodadvances.2018018093 29898879 PMC6020817

[134] Ballabio E Milne TA. Molecular and epigenetic mechanisms of MLL in human leukemogenesis. Cancers. (2012) 4:904–44. 10.3390/cancers4030904 24213472 PMC3712720

[135] Takahashi S Yokoyama A. The molecular functions of common and atypical MLL fusion protein complexes. Biochim Biophys Acta Gene Regul Mech. (2020) 1863:194548. 10.1016/j.bbagrm.2020.194548 32320750

[136] Rice S Jackson T Crump NT Fordham N Elliott N O'Byrne S. A novel human fetal liver-derived model reveals that MLL-AF4 drives a distinct fetal gene expression program in infant ALL. bioRxiv. (2020):2020.11.15.379990. 10.1101/2020.11.15.379990. [Epub ahead of print].

[137] Mullighan CG Goorha S Radtke I Miller CB Coustan-Smith E Dalton JD. Genome-wide analysis of genetic alterations in acute lymphoblastic leukaemia. Nature. (2007) 446:758–64. 10.1038/nature05690 17344859

[138] Kuiper RP Schoenmakers EF van Reijmersdal SV Hehir-Kwa JY van Kessel AG van Leeuwen FN. High-resolution genomic profiling of childhood ALL reveals novel recurrent genetic lesions affecting pathways involved in lymphocyte differentiation and cell cycle progression. Leukemia. (2007) 21:1258–66. 10.1038/sj.leu.2404691 17443227

[139] Papaemmanuil E Rapado I Li Y Potter NE Wedge DC Tubio J. RAG-mediated recombination is the predominant driver of oncogenic rearrangement in ETV6-RUNX1 acute lymphoblastic leukemia. Nat Genet. (2014) 46:116–25. 10.1182/blood.V122.21.807.807 24413735 PMC3960636

[140] Shi Y Su XB He KY Wu BH Zhang BY Han ZG. Chromatin accessibility contributes to simultaneous mutations of cancer genes. Sci Rep. (2016) 6:35270. 10.1038/srep35270 27762310 PMC5071887

[141] Rowe RG Lummertz da Rocha E Sousa P Missios P Morse M Marion W. The developmental stage of the hematopoietic niche regulates lineage in MLL-rearranged leukemia. J Exp Med. (2019) 216:527–38. 10.1084/jem.20181765 30728174 PMC6400531

